# Design features and elemental/metal analysis of the atomizers in pod-style electronic cigarettes

**DOI:** 10.1371/journal.pone.0248127

**Published:** 2021-03-09

**Authors:** Esther E. Omaiye, Monique Williams, Krassimir N. Bozhilov, Prue Talbot

**Affiliations:** 1 Environmental Toxicology Graduate Program, University of California Riverside, Riverside, CA, United States of America; 2 Department of Molecular, Cell and Systems Biology, University of California Riverside, Riverside, CA, United States of America; 3 Central Facility for Advanced Microscopy and Microanalysis, University of California Riverside, Riverside, CA, United States of America; Battelle Memorial Institute, UNITED STATES

## Abstract

**Background:**

The atomizers of electronic cigarettes (ECs) contain metals that transfer to the aerosol upon heating and may present health hazards. This study analyzed 4^th^-generation EC pod atomizer design features and characterized their elemental/metal composition.

**Methods:**

Eleven EC pods from six brands/manufacturers were purchased at local shops and online. Pods were dissected and imaged using a Canon EOS Rebel SL2 camera. Elemental analysis and mapping of atomizer components was done using a scanning electron microscope coupled with an energy dispersive x-ray spectrometer.

**Results:**

EC pods varied in size and design. The internal atomizer components were similar across brands except for variations occurring mainly in the wicks and filaments of some products. The filaments were either Elinvar (nickel, iron, and chromium) (36.4%), nichrome (36.4%), iron-chromium (18.2%), or nickel (9%). Thick wires present in 55% of the atomizers were mainly nickel and were joined to filaments by brazing. Wire-connector joints were Elinvar. Metal air tubes were made of Elinvar (50%), nickel, zinc, copper, and tin (37.5%), and nickel and copper (12.5%). Most of the wick components were silica, except for two pods (PHIX and Mico), which were mainly ceramic. Connectors contained gold-plated nickel, iron-chromium multiple alloys of nickel, zinc, gold, iron, and copper. Wick chambers were made of Elinvar. Outer casings were either nickel, copper-tin, or nickel-copper alloys. Magnets were nickel with minor iron, copper, and sulfur. Some frequently occurring elements were high in relative abundance in atomizer components.

**Conclusions:**

The atomizers of pods are similar to previous generations, with the introduction of ceramic wicks and magnets in the newer generations. The elements in EC atomizers may transfer into aerosols and adversely affect health and accumulate in the environment.

## Introduction

The external appearance, design, battery power, atomizers, and nicotine delivery of electronic cigarettes (ECs) have evolved over the last decade, with four generations recognized [1–3]. Most first-generation or cig-a-like/cartomizer products (e.g., NJOY, V2 Cigs, BluCig, Mark Ten, and Vuse) are similar in size and resemblance to tobacco cigarettes. They contain an atomizer designed to produce an aerosol by heating e-liquids rather than burning tobacco [4–6]. Second-generation ECs or clearomizers (e.g., Ego C Twist) have larger atomizers/tanks with some models (e.g., Vuse) lacking solder joints, polyfil fibers, and microprocessors. Larger fluid reservoirs and batteries are used in the third-generation products (mods), such as iTaste MVP, Smok Alien, and iPV6X [1]. Third generation ECs generally lack thick wires, fibers, and sheaths and are user-friendly with variable power settings.

Fourth-generation ECs or pods, which now comprise a significant share of the EC market [7–9], have relatively low-powered batteries, an e-liquid reservoir, and an atomizer/mouthpiece. Pods can be prefilled (closed-system), refillable (open-system), or disposable. Pod fluids differ from prior generation fluids in that they contain high concentrations of nicotine (~ 50–60 mg/mL) [2] and acid, which protonates the nicotine and makes the aerosol less harsh [10]. The combination of high nicotine delivered in an acidic aerosol may increase the possibility of addiction of novice users [10, 11].

Potentially harmful elements/metals, including nickel, lead, cadmium, arsenic, and chromium, are in atomizers of the first three EC generations [6, 12, 13] and vape fluids [14–19]. Upon heating, elements/metals in vape fluids can transfer into aerosols and increase in concentrations in the fluid after vaping [18, 19]. The concentration of these elements in aerosols varies with EC products, and it is usually higher in third-generation products, which operate at higher power [12, 13, 18–21].

Despite the ability of harmful metals to transfer into EC fluids and aerosols, extensive reviews of current literature on the health effects of ECs have presented little information on the impact of inhaled elements on users [22–24]. Fourth-generation ECs remain very popular with high school students and adolescents and dominate the current EC market [25, 26]. However, we have very little knowledge about the elemental composition of their atomizers. Our goals for this study were to: (1) characterize the atomizer components and design features of popular pod ECs, and (2) identify and map the elements/metals in their atomizers.

## Materials and methods

### Selection and purchase of EC pod devices

In 2019, popular prefilled and refillable pod EC products were identified in multiple reviews on vape forums, blogs, company websites, and then purchased online from the manufacturer’s website or vape shops from reputable third-party vendors. The products selected for evaluation were PHIX (ECS Global LLC., Los Angeles, CA), Kilo 1K (Kilo E-liquid Inc., La Mirada, CA), KWIT Stick (Aspire., USA), Suorin Air (Shenzhen Blumark Technology Co., Ltd, China), Suorin Drop (Shenzhen Blumark Technology Co., Ltd, China), Suorin Edge (Shenzhen Blumark Technology Co., Ltd, China), SMOK Mico (Shenzhen IVPS Technology CO., Ltd, China), SMOK Infinix (Shenzhen Blumark Technology Co., Ltd, China), and SMOK NORD (Shenzhen Blumark technology Co., Ltd, China). Two replacement coils were evaluated within the SMOK NORD brand, a 1.4-ohm coil filament designed for mouth-to-lung (MTL) vaping and a 0.6-ohm mesh filament for sub-ohm vaping. Upon receipt, all products were inventoried and stored at room temperature until analyzed. Multiple EC devices and pods from the same brand were purchased simultaneously to ensure that the analysis was performed on products from the same purchase batch.

### Dissection, scanning electron microscopy, and elemental analysis of EC pod atomizers

Prefilled (closed system) EC pods were emptied of the liquid. The atomizers of all pods were then carefully dissected to expose the internal components of interest. Each component was then photographed using a Canon EOS Rebel SL2.

The dissected components were mounted on aluminum pin stubs covered with conductive carbon tape to prevent charging during the analysis [5]. The edges of any plastic components were covered with carbon conductive paint to minimize charging under the electron beam during SEM imaging. Morphological and elemental analyses were performed using a Thermo Fisher Scientific Co. NovaNano-SEM 450 Scanning Electron Microscope (SEM) equipped with an Oxford Instruments Inc. energy dispersive X-ray spectrometer (EDS) fitted with an X-Max50 50 mm^2^ SDD detector with an energy resolution of 126 eV at MnK-α located at the Central Facility for Advanced Microscopy and Microanalysis at the University of California at Riverside. SEM images were obtained using a secondary electron mode with a dedicated detector at 15 kV. EDS spectra and elemental maps were acquired and processed with the Oxford Instruments Inc. Aztec Synergy v.4 software package to qualitatively reveal the distribution of chemical elements within the sample area. Elemental identification is based on the system’s ability to identify and differentiate specific element peaks with an atomic number of 5 or higher on the spectra. The detection limit for the EDS method is about 0.1 wt. %. For ease and clarity of data analysis, we have set an arbitrary threshold value of 5 wt. %. Elements present above the > 5% threshold are denoted as “major.” Those below the threshold are “minor.” Quantification of the elements was performed by processing the acquired EDS spectra using the standard-less routine and Oxford Instruments Inc. factory-supplied table of elemental standards incorporated in the Aztec software.

## Results

### Design and components of prefilled and refillable pod ECs

**Fig 1:**
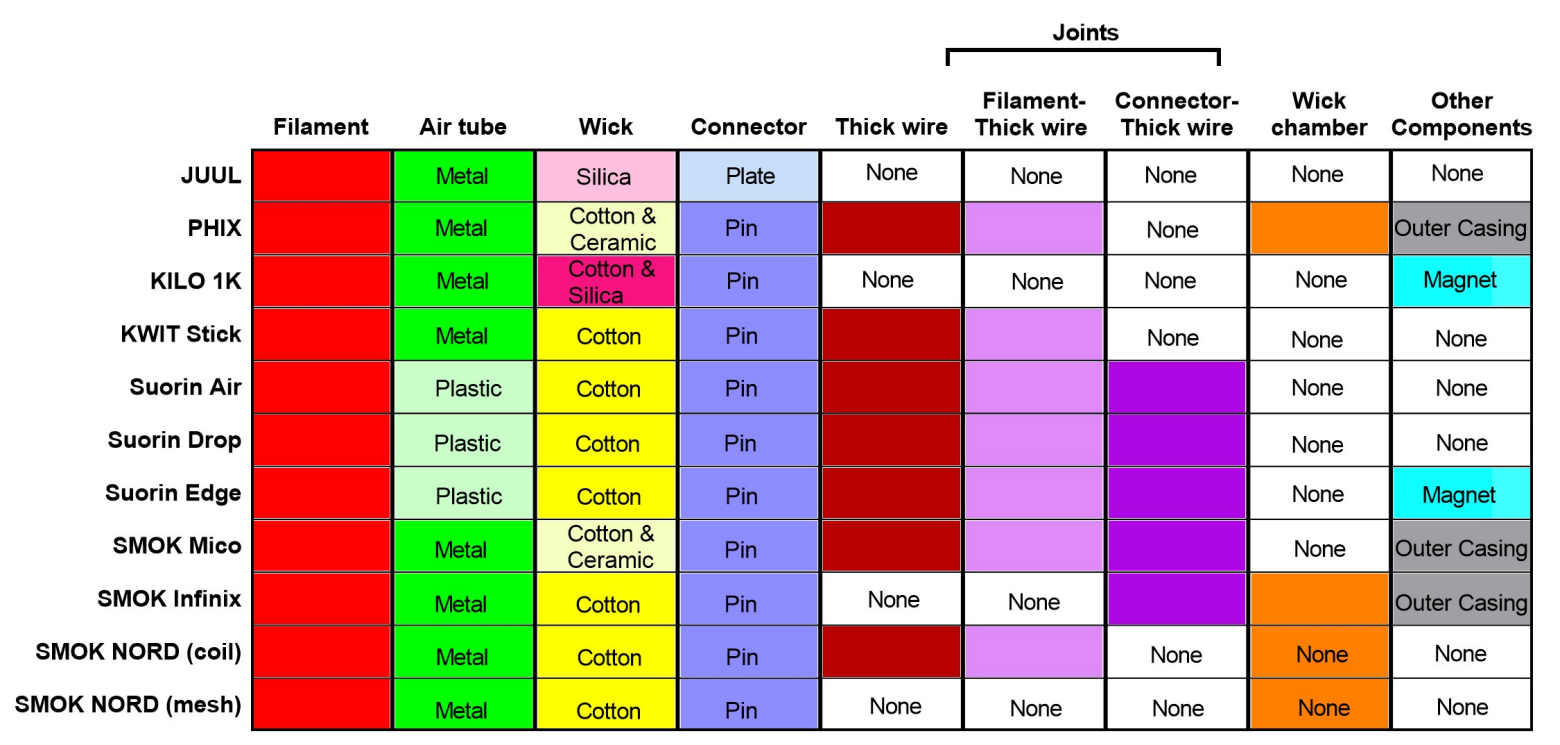
Atomizer components that were present in multiple brands of pod ECs. ECs are listed on the left axis, and pod components are listed above each column.

The design and pod components of 11 products purchased in 2019 were compared (S1 Fig and Fig 1). Pod design shapes included rectangle, diamond, square, and teardrop shapes. Discreet airholes were located at different sites on each device. (S1 Fig). The rectangular or duck-billed shape mouthpieces were located close to the reservoir, which held 0.7–3 ml of fluid. Generally, low volume reservoirs were present in closed-pod systems, while higher volume reservoirs were in the refillable and open-system pods.

All products had filaments, which are required for generating aerosols. Most products had metal air tubes; however, the air tube was plastic in three products. The wicks were either cotton, silica, or ceramic. Some products had two wicks: cotton and ceramic (yellow and magenta for PHIX) or cotton and silica (yellow and light pink for Kilo 1K) (Fig 1). A connector pin was present in all pods except JUUL™, which had a connector plate. The thick wire was either brazed to the filament or joined to the connector component. (Fig 1). Four products contained a wick chamber, which held the wick in place. A magnet, which secured the pod to the battery, was present in three products (Fig 1).

**Fig 2:**
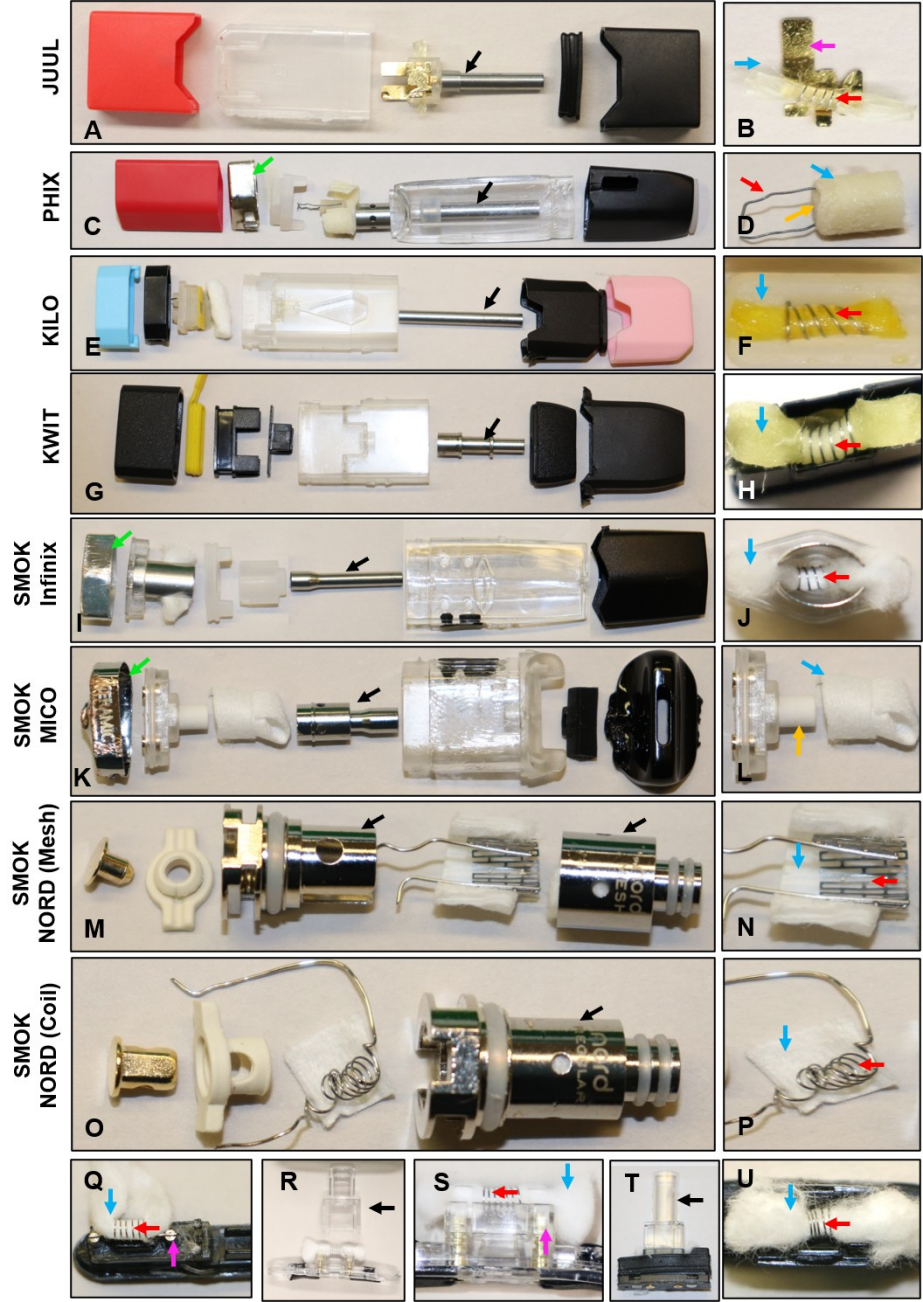
Anatomy and internal design of atomizers from pod ECs. JUUL™ (A-B), PHIX (C-D), Kilo 1K (E-F), KWIT Stick (G-H), SMOK Infinix (I-J) SMOK Mico (K-L), SMOK NORD 0.6-ohm (mesh) filament (M-N), SMOK NORD 1.4-ohm (coil) filament (O-P), Suorin Air (Q), Suorin Drop (R-S) Suorin Edge (T-U). JUUL™, PHIX, and Kilo 1K are prefilled pods. KWIT Stick is a prefilled and refillable pod. SMOK and Suorin pods are refillable. Specific key components are indicated by colored arrows: air tube = black; cotton/silica wick = blue; ceramic wick = orange; filament and thick wires = red; connector plate/pin = pink; outer casing = green.

The design and layout of the atomizer components varied among products (Fig 2). An air tube (metal or plastic) (black arrows), filament (red arrows), and a wick (cotton or silicon) (blue arrows) were present in all brands. SMOK Mico and PHIX had additional ceramic wicks (orange arrows) (Fig 2D and 2L). The connector plate/pins (pink arrows in Fig 2B, 2Q and 2S) located at the base of all pods provided a path for the current to flow from the atomizer’s battery. All pods with a thick wire had a filament-wire joint, a wire-connector joint, or both. All Suorin products had plastic air tubes and large cotton wicks (Fig 2Q–2U). PHIX, SMOK (Infinix, and NORD) had a chamber that housed the wick and filament (Fig 2C, 2M and 2O). In SMOK NORD, this chamber served the same purpose as the air tube. A magnet, which secures the pod to the battery when the device is in use, was present in the Kilo 1K, Suorin, and SMOK brands. An outer casing (green arrow), which held the atomizer components together, was present in PHIX and SMOK (Infinix and Mico) pods (Fig 2C, 2I and 2K).

### Elemental analysis of pod EC atomizers

**Fig 3:**
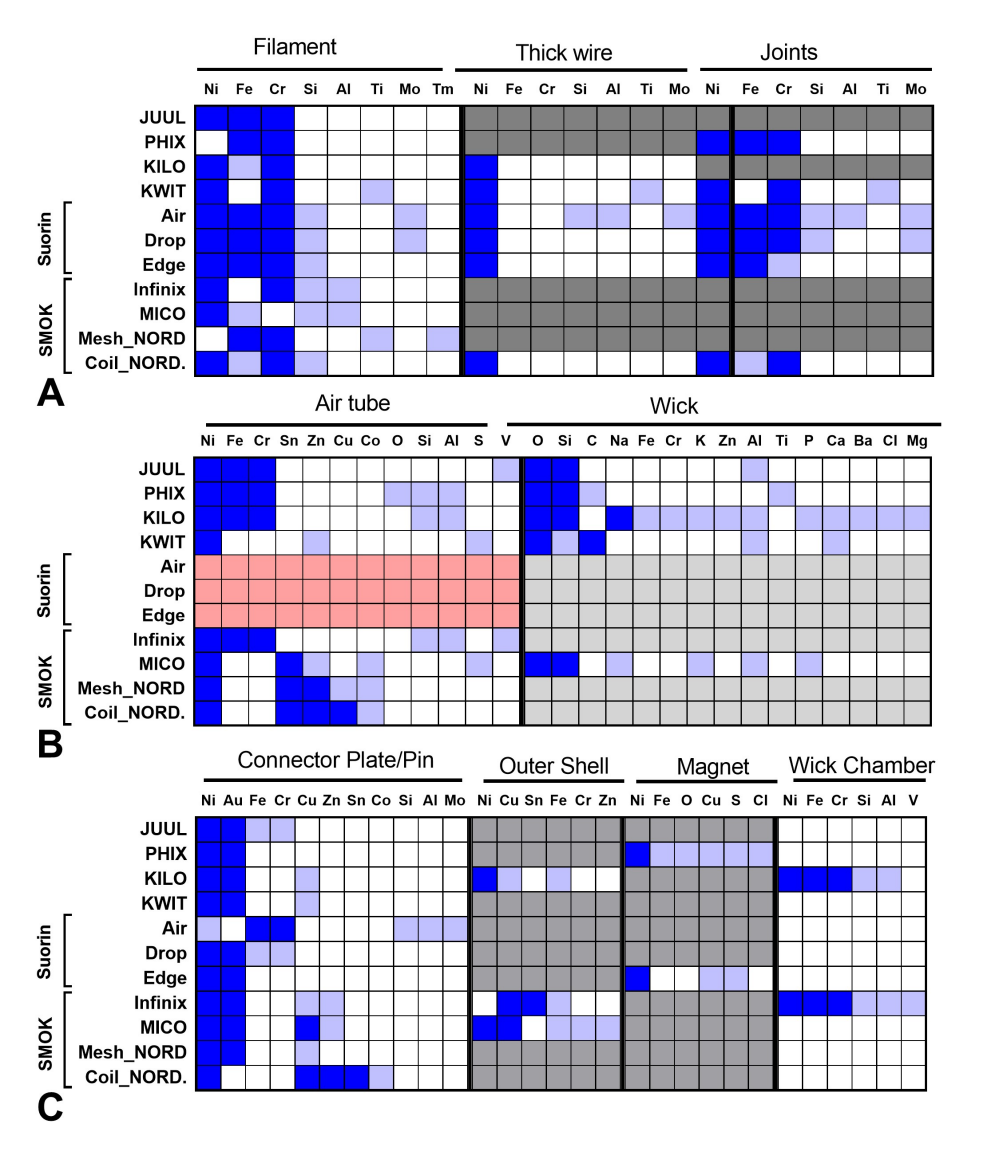
Heat maps showing the elemental composition of atomizer components in pod ECs. Blue squares = elements that were major peaks in the EDS spectra. Light blue squares = elements that were minor peaks in spectra. Dark gray squares = components that were not present. Light gray squares = components that were present but not analyzed because they were identical to other components. Pink squares = components that were made of plastic and not analyzed.

**Fig 4:**
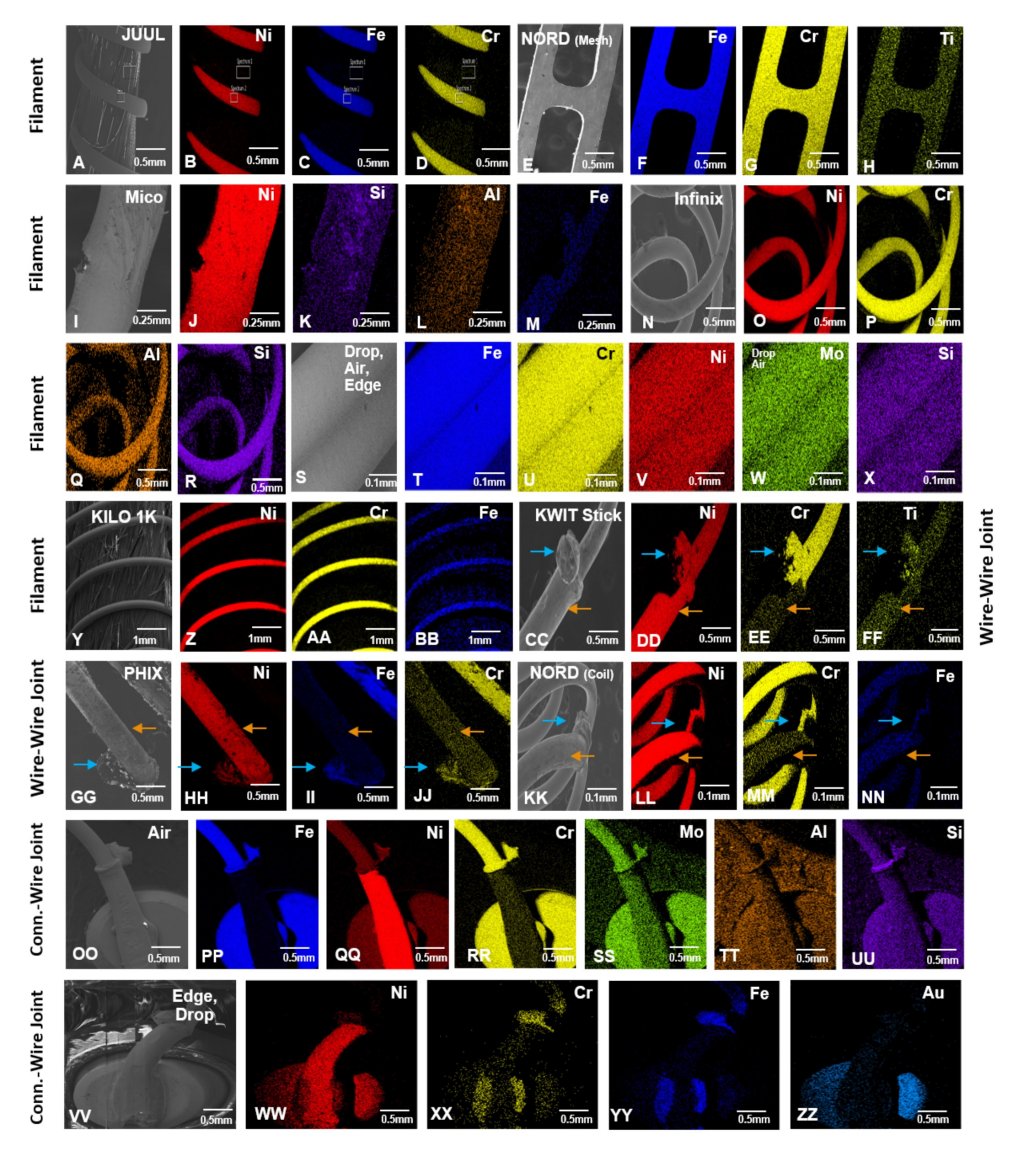
SEM images and EDS elemental maps of the filaments and joints from pod EC atomizers. For filaments, JUUL™ (A) was made of nickel (B), iron (C), and chromium (D). SMOK NORD (mesh) (E) was made of iron (F), chromium (G), and titanium (H). SMOK Mico (I) was made of nickel (J), silicon (K), aluminum (L), and iron (M). SMOK Infinix (N) was made of nickel (O), chromium (P), aluminum (Q), and silicon (R). Suorin Drop, Air, and Edge (S) were made of iron (T), chromium (U), nickel (V), molybdenum (W), and silicon (X). Kilo 1K (Y) was made of nickel (Z), chromium (AA), and iron (BB). KWIT Stick (CC) was made of nickel (DD), chromium (EE), and titanium (FF). For filament-wire joints, KWIT Stick (CC) was made of nickel (DD) and chromium (EE). PHIX (GG) was made of nickel (HH), iron (II), and chromium (JJ). SMOK NORD (coil) (KK) was made of nickel (LL), chromium (MM), and iron (NN). Suorin Air (OO) was made of iron (PP), chromium (RR), molybdenum (SS), aluminum (TT), and silicon (UU). Suorin Edge and Drop (VV) were made of chromium (XX) and iron (YY). For connector-wire joints, Suorin Air (OO) was made of a droplet of iron (PP), nickel (QQ), chromium (RR), molybdenum (SS), aluminum (TT), and silicon (UU). Suorin Edge and Drop (VV) were made of nickel (WW), chromium (XX), and iron (YY). Orange arrows in CC–NN show thick wires, while blue arrows show joints between the thick wire and filament.

**Fig 5:**
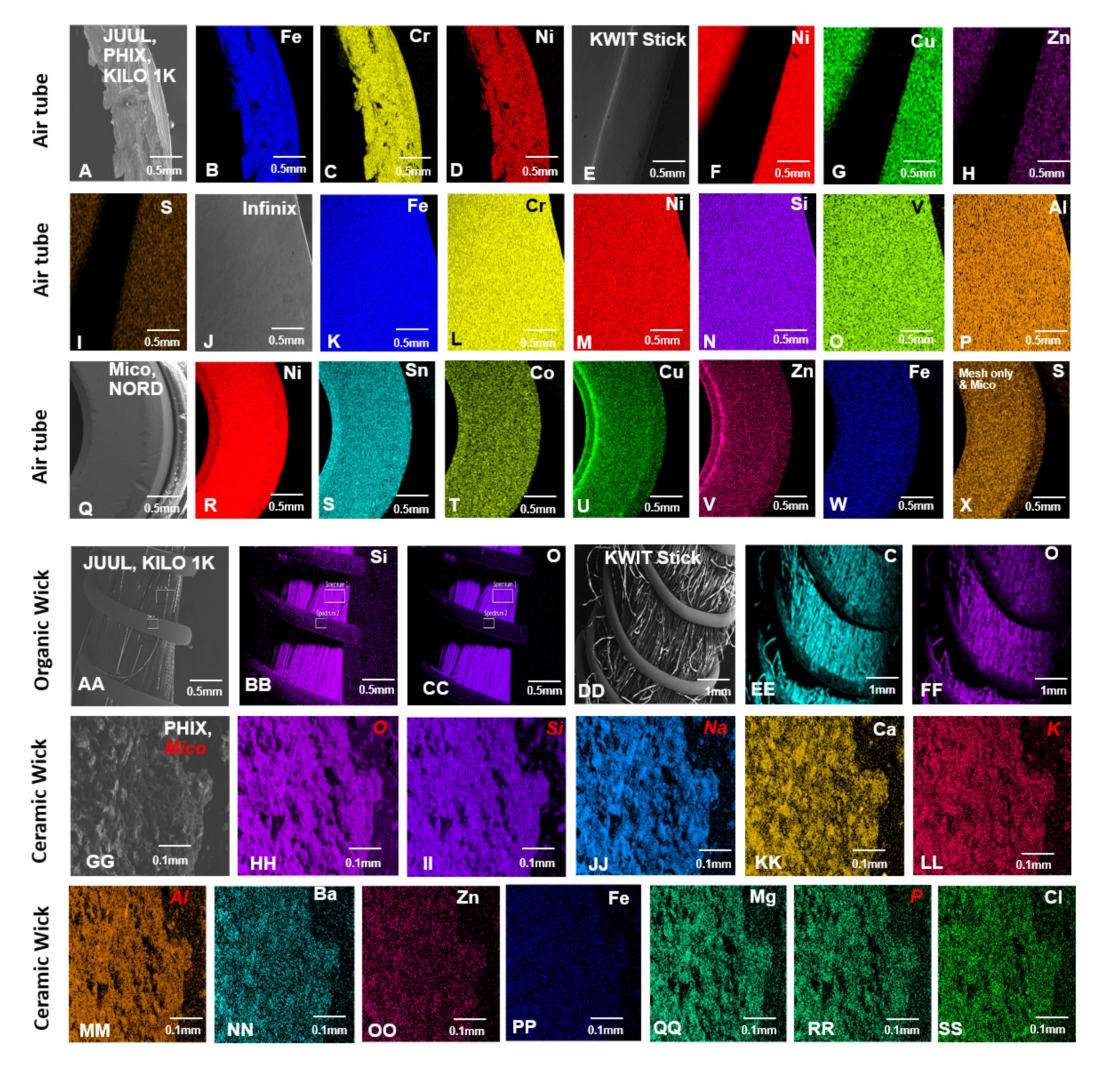
SEM images and EDS elemental maps of the air tubes and wicks from pod EC atomizers. JUUL™, PHIX, and Kilo 1K (A) were made of iron (B), chromium (C), and nickel (D). KWIT Stick (E) was made of nickel (F), copper (G), zinc (H), and sulfur (I). SMOK Infinix (J) was made of iron (K), chromium (L), nickel (M), silicon (N), vanadium (O), and aluminum (P). SMOK Mico and NORD (Q) were made of nickel (R), tin (S), cobalt (T), copper (U), zinc (V), iron (W), and sulfur (X). Organic wicks in JUUL™ and Kilo 1K (AA) were made of silicon (BB) and oxygen (CC), and KWIT (DD) was made of carbon (EE) and oxygen (FF). PHIX and SMOK Mico (GG) had ceramic wicks consisting of iron (HH), silicon (II), sodium (JJ), calcium (KK), potassium (LL), aluminum (MM), barium (NN), zinc (OO), iron (PP), magnesium (QQ), phosphorus (RR), and chlorine (SS). Elements highlighted in red for ceramic wicks were present only in SMOK Mico.

**Fig 6:**
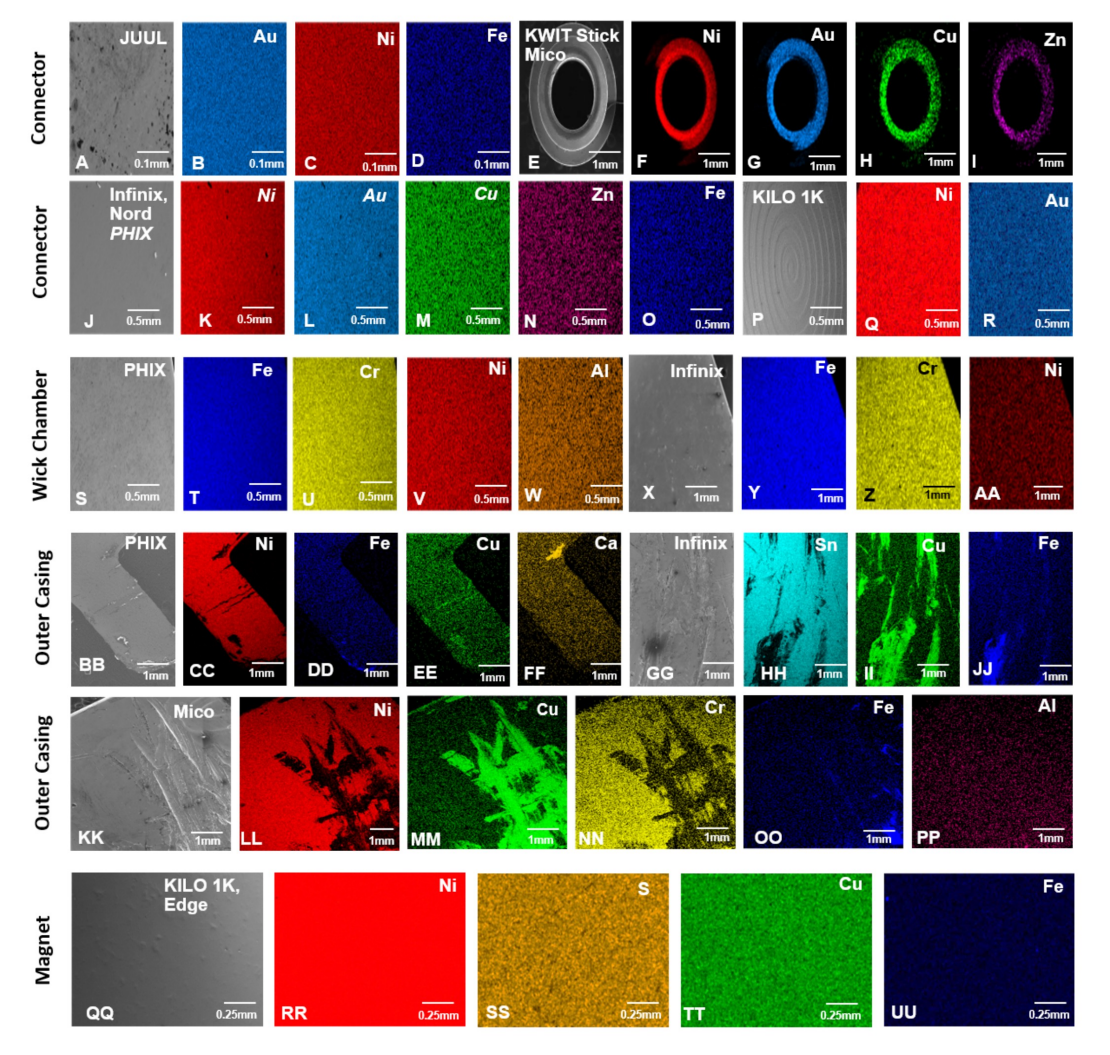
SEM images and EDS elemental maps of miscellaneous components from pod EC atomizers. Connectors present in JUUL™ (A) were made of gold (B), nickel (C), and iron (D). KWIT Stick and SMOK Mico (E) were made of nickel (F), gold (G), copper (H), and zinc (I). SMOK Infinix, SMOK NORD, and PHIX (J) were made of nickel (K), gold (L), copper (M), zinc (N), and iron (O). Kilo 1K (P) was made of nickel (Q) and gold (R). Wick chambers present in PHIX (S) was made of iron (T), chromium (U), nickel (V), and aluminum (W). SMOK Infinix (X) was made of iron (Y), chromium (Z), and nickel (AA). The outer casing present in PHIX (BB) was made of nickel (CC), iron (DD), copper (EE), and calcium (FF). SMOK Infinix (GG) was made of tin (HH), copper (II), and iron (JJ). SMOK Mico (KK) was made of nickel (LL), copper (MM), chromium (NN), iron (OO), and aluminum (PP). Magnet components present in Kilo 1K and Suorin Edge (QQ) were made of nickel (RR), sulfur (SS), copper (TT), and iron (UU).

The elemental composition of 11 pod ECs was analyzed using SEM and EDS (Figs 3–6, Table 1, and S2–S5 Figs). Examples of the major (silicon, oxygen, gold, and nickel) and minor (aluminum, chromium, and iron) peaks are shown in EDS spectra for the wick and connector plate components of JUUL™ atomizers (S2 Fig). The relative abundance of elements based on the EDS spectra for each atomizer component is summarized in Fig 3. The blue and light blue squares are elements with major and minor abundance, respectively. Pink squares are components made of plastic. Dark gray squares indicate components absent in atomizers, and light gray squares represent components that were present but not analyzed. Due to similar organic materials being used in components such as the wick, only selected pods were evaluated in the SEM. SEM images of all components and their corresponding elemental maps arranged from left to right based on high relative abundance are shown in Figs 4–6, and S3–S5 Figs.

**Table 1:** Summary of element/metal analysis of pod EC atomizer components.

Brand	Product	Filament	Thick Wire	Joints	Air tube	Wick	Connector Plate/Pin	Miscellaneous
**Pax Labs**	JUUL™	Ni, Fe, Cr	None	None	Ni, Fe, Cr (*V*)	O, Si	Ni, Fe, Au (*Cr*)	
**Kilo E-liquid Inc.**	KILO 1K	Ni, Cr (*Fe*)	None	None	Ni, Fe, Cr	O, Si (*C*, *Ti*)	Ni, Au	
**Aspire**	KWIT Stick	Ni, Cr (*Ti*)	Ni (*Al*, *Ti*)	Ni, Cr (*Ti*)	Ni, Cu (*Zn*, *S*)	O, C (Si, Al, Ca)	Ni, Zn, Au (*Cu*)	**Outer shell:** Ni (*Fe*, *Cu*, *Ca*)
**ECS Global LLC**	PHIX	Fe, Cr	Ni	Ni, Fe, Cr	Ni, Fe, Cr	O, Si, Na, (*Fe*, *K*, *Zn*, *Al*, *P*, *Ca*, *Ba*, *Cl Mg*)	Ni, Au (*Cu*)	**Wick Chamber:** Ni, Fe, Cr (*Al*). **Outer shell:** Ni (*Fe*, *Cu*, *S*)
**SMOK**	Infinix	Ni, Cr (*Si*, *Al*)	None	None	Ni, Fe, Cr (*Si*, *Al*, *V*)		Ni, Au (*Si*, *Zn*, *Al*, *Cu*)	**Wick Chamber:** Ni, Fe, Cr (*Al*).
								**Outer shell:** Cu, Sn (*Fe*)
**SMOK**	MICO	Ni (*Fe*, *Si*, *Al*)			Ni, Zn, Cu, Sn (*Fe*, *Co*, *S*)	O, Si (*K*, *Al*, *Na*, *P*)	Ni, Au, Cu (*Zn*)	**Outer shell:** Ni, Cu (minor: Fe, Cr, Al).
**SMOK.**	NORD (coil).	Ni, Cr (*Fe*, *Si*)	Ni	Ni, Cr (*Fe*)	Ni, Zn, Cu, Sn (*Fe*, *Co*)		Ni, Zn, Au, Cu (*Fe*)	
**SMOK**	NORD (mesh)	Fe, Cr (*Ti*)	None	None	Ni, Zn, Cu, Sn (*Fe*, *Co*, *S*)		Ni, Zn, Au (*Fe*, *Cu*)	
**Suorin**	Air	Ni, Fe, Cr (*Si*, *Mo*)	Ni (*Si*, *Al*, *Mo*)	Ni, Fe, Cr (*Si*, *Al*, *Mo*)	None		Fe, Cr (*Ni*, *Si*, *Al*, *Mo*)	
**Suorin**	Edge	Ni, Fe, Cr (*Si*)	Ni	Ni, Fe, Cr	None		Ni, Au	**Magnet:** Ni (*Cu*, *S*)
**Suorin**	Drop	Ni, Fe, Cr (*Si*, *Mo*)	Ni	Ni, Fe, Cr	None		Ni, Au (*Fe*, *Cr*)	

Full names of elements. Ni = Nickel, Fe = iron, Cr = chromium, Ti = Titanium, Si = silicon, Al = aluminum, Mo = molybdenum, V = Vanadium, Cu = copper, Zn = zinc, S = sulfur, Sn = tin, Co = cobalt, O = oxygen, Si = silicon, C = carbon, Ca = calcium, Na = sodium, K = potassium, P = phosphorus, Ca = calcium, Ba = barium, Cl = chlorine, Mg = magnesium, Au = gold. Elements *italicized* indicate minor relative abundance.

Nickel, iron, and chromium alloys in the form of Elinvar (nickel, iron, chromium alloy) (36.4%), nichrome (nickel and chromium) (36.4%), stainless steel (iron and chromium) (18.2%), or nickel (9.1%) were the most abundant elements in the filaments (Figs 3A and 4A-4FF). Some filaments also contained minor amounts of silicon, aluminum, titanium, and molybdenum. Within the SMOK (NORD, Mico, and Infinix) brand, filament composition and structure varied between products. While the 0.6-ohm NORD mesh filament was mainly iron and chromium (Figs 3A and 4E-4H), the Mico was mainly nickel with minor amounts of silicon, aluminum, and iron (Fig 4I–4M). The Infinix and the 1.4-ohm NORD coil filaments were mainly nichrome (Fig 4N–4P and 4KK-4NN). The filaments in the Suorin (Air, Edge, and Drop) pods contained iron, chromium, nickel, and silicon, with molybdenum present only in the Suorin Drop and Suorin Air filament (Fig 4S–4X and S3 Fig).

When present, the thick wire brazed to the filament was predominantly nickel with minor amounts of silicon, aluminum, titanium, and molybdenum in some products (Figs 3A and 4CC–4ZZ, Table 1, and S3 Fig). Connector-wire joints contained major metals found in the thick wires and minor elements such as molybdenum, aluminum, and silicon (Fig 4OO–4ZZ and S3 Fig).

Except for the plastic air tubes in the Suorin pods (Fig 3B), air tubes were either Elinvar, nickel, or nickel alloy containing tin, zinc, or copper as major elements (Figs 3B and 5A–5X, S4 Fig). Minor amounts of zinc, copper, cobalt, oxygen, silicon, aluminum, sulfur, and vanadium were also present in some air tubes (Fig 3B). A nickel-coated brass (copper, zinc) air tube with little sulfur was found in KWIT Stick (Figs 3B and 5E-5I). Within the SMOK brand, air tube composition varied in the NORD, Mico, and Infinix. While the Infinix air tube was mainly Elinvar, the Mico was majorly nickel and tin (Figs 3B and 5J–5X). The NORD variants were almost identical except for a higher abundance of copper in the pod with the 1.4-ohm NORD coil filament.

The wicks in pod atomizers were mainly oxygen and silicon. However, PHIX and KWIT Stick wicks also had significant amounts of sodium and carbon, respectively (Figs 3B and 5AA–5SS, S4 Fig). Wicks in some products contained minor amounts of iron, chromium, potassium, zinc, aluminum, titanium, phosphorus, calcium, barium, chlorine, or magnesium. Most of the minor elements were present in the ceramic wicks, which had the heating coils embedded in them (Figs 3B and 5GG–5SS, S4 Fig). Since all samples were prepared in the same manner, it is unlikely the carbon or the minor elements were from residual fluid, which would have been present on all samples and other components.

Most connector components (plate/pins) were mainly gold-plated nickel (Figs 3C and 6A-6R). Additional elements, including iron, chromium, zinc, and copper, were also in high abundance in several brands (Fig 3C). Lower abundance elements in connector components included chromium, copper, zinc, tin, cobalt, silicon, aluminum, and molybdenum. A connector plate, which was present only in JUUL™, was comprised of nickel, iron, and gold with minor chromium (Figs 1, 2B and 3C, S5 Fig).

Miscellaneous components found in some products included a wick chamber, outer casing, and magnet. The wick chambers in PHIX and SMOK Infinix were mainly Elinvar with minor aluminum (Fig 6S–6AA). The outer casing in PHIX and SMOK (Infinix and Mico) was mainly nickel, copper, and tin (Figs 3C and 6BB-6PP). Minor levels of iron, chromium, aluminum, and calcium were also present. Magnets were mainly nickel with minor iron, copper, and sulfur (Figs 3C and 6QQ–6UU).

### Frequency of occurrence of elements in atomizers of fourth generation pod ECs

**Fig 7:**
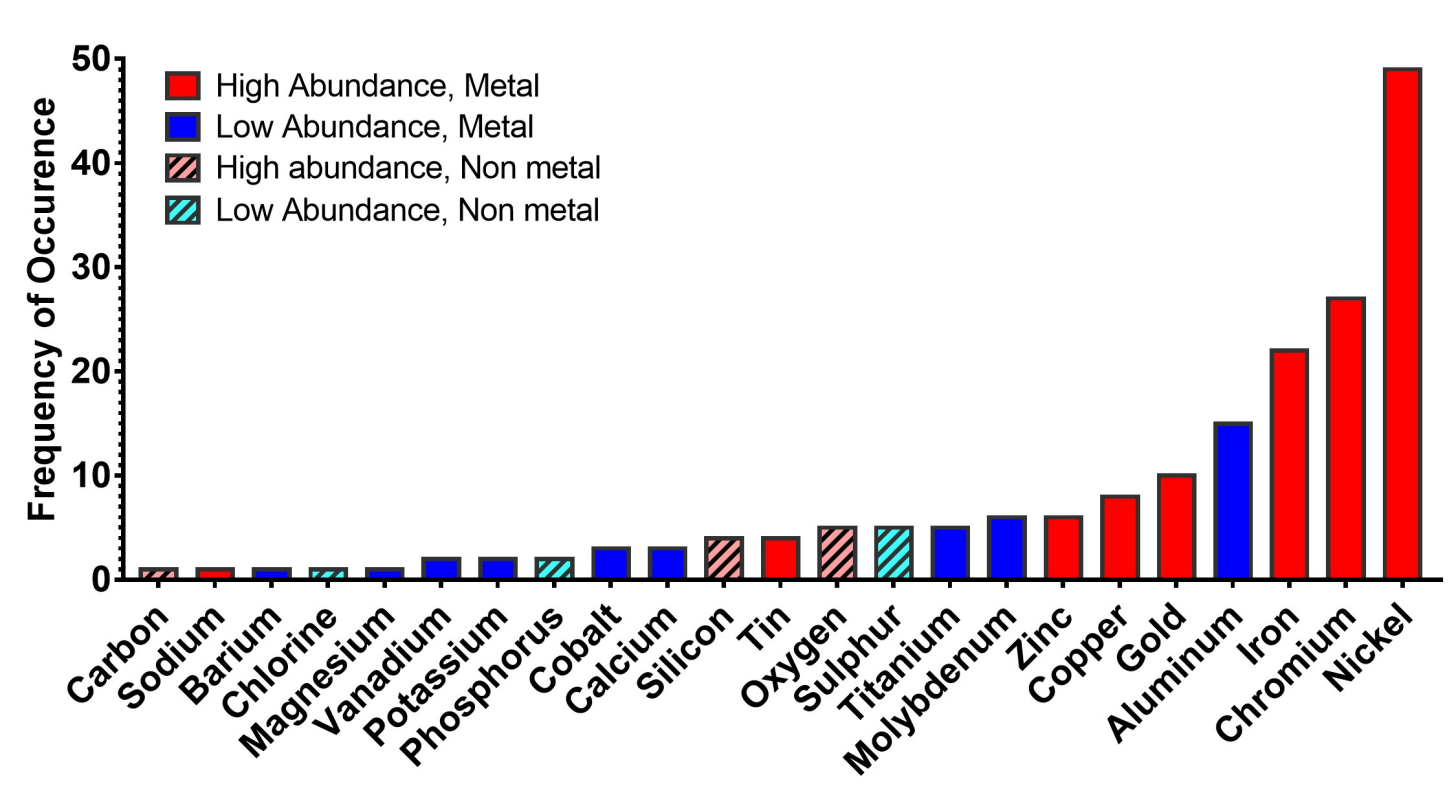
Frequency of occurrence of elements in pod EC atomizers. The x-axis shows metal/elements sorted according to increasing frequency of their occurrence (1–49), with nickel being the highest. Bars are coded based on each element’s relative abundance. Solid bars are metals, while hatched bars are non-metals. Red bars = metals in major abundance, blue bars = metals in minor abundance, red hatched bars = non-metals in major abundance, hatched blue bars = non-metals in minor abundance.

Information based on the relative abundances of elements in our study (Fig 3) was used to evaluate the frequency of 23 metals/elements in pod atomizer components (Fig 7). The nine most frequently found metals that appeared in 5 or more components in descending order of frequency were: nickel, chromium, iron, aluminum, gold, copper, zinc, molybdenum, titanium. All except aluminum, molybdenum, and titanium were in relatively high abundance (blue bars in Fig 7).

### Comparison of atomizer components from fourth generation pod ECs

**Fig 8:**
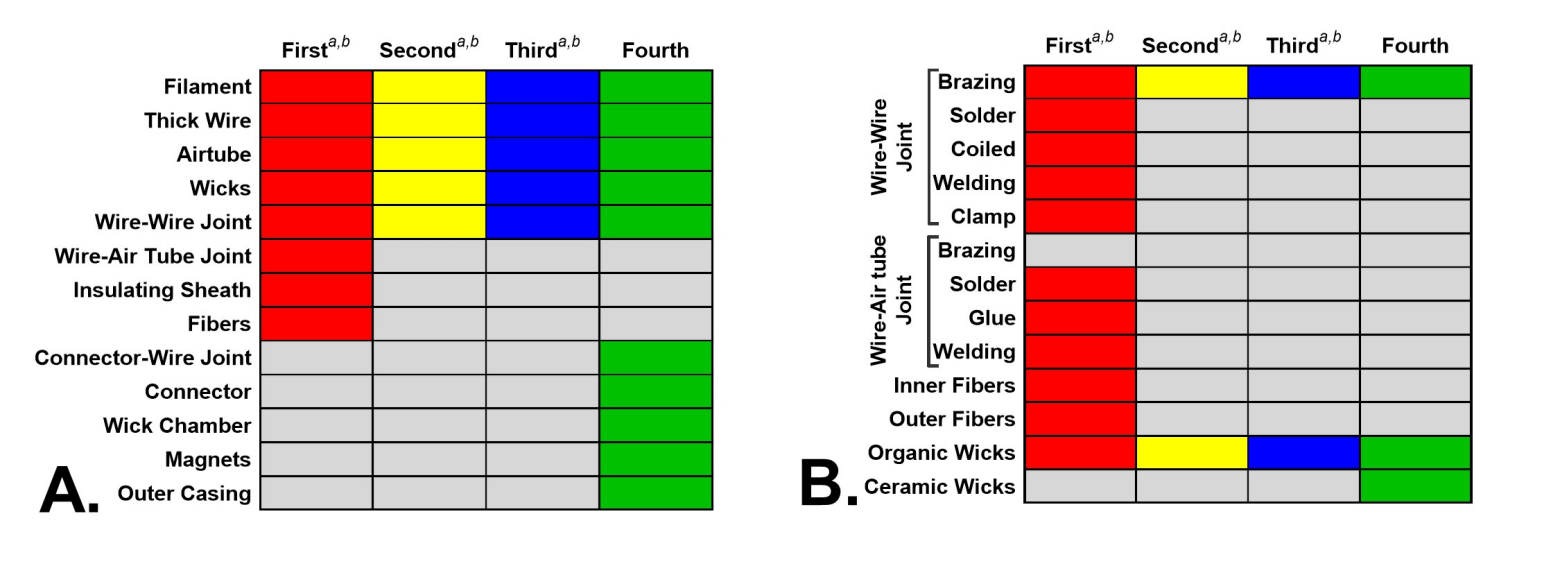
Atomizer components, methods of joining, and types of component in four EC generations. (A) Atomizer components in four EC generations. (B) Types of joining methods, fibers, and wicks in four EC generations. The columns indicate the four EC generations (first = cartomizers/disposables, second = clearomizers/tanks, third = mods, fourth = pods). The y-axis lists major atomizer components. ^a^ = Williams et al. 2019a; ^b^ = Williams et al. 2019b.

The components and method of joining components in atomizers of previous generations of ECs [1, 6] were compared to the fourth generation ECs (Fig 8). Filament, thick wire, air tube, wick, and wire-wire joint were present and preserved across all EC generations (Fig 8A). Wire-air tube joint, sheath, and fiber were components found only in first-generation ECs. Consequently, connectors, connector-wire joint, wick chamber, magnet, and outer casing are evolving components that were present only in the fourth generation ECs (Fig 8A).

Within atomizers, brazing was used in all four generations for wire-wire joining. Soldering, coiling, welding, and clamping were used only in the earliest EC products (Fig 8B). Wire-air tube joints were only in the first generation, where all joining types except brazing were used. Both inner and outer fibers were present only in the first generation, and ceramic wicks were found only in the fourth generation (Fig 8B).

### Elements in atomizer components across multiple EC generations

**Fig 9:**
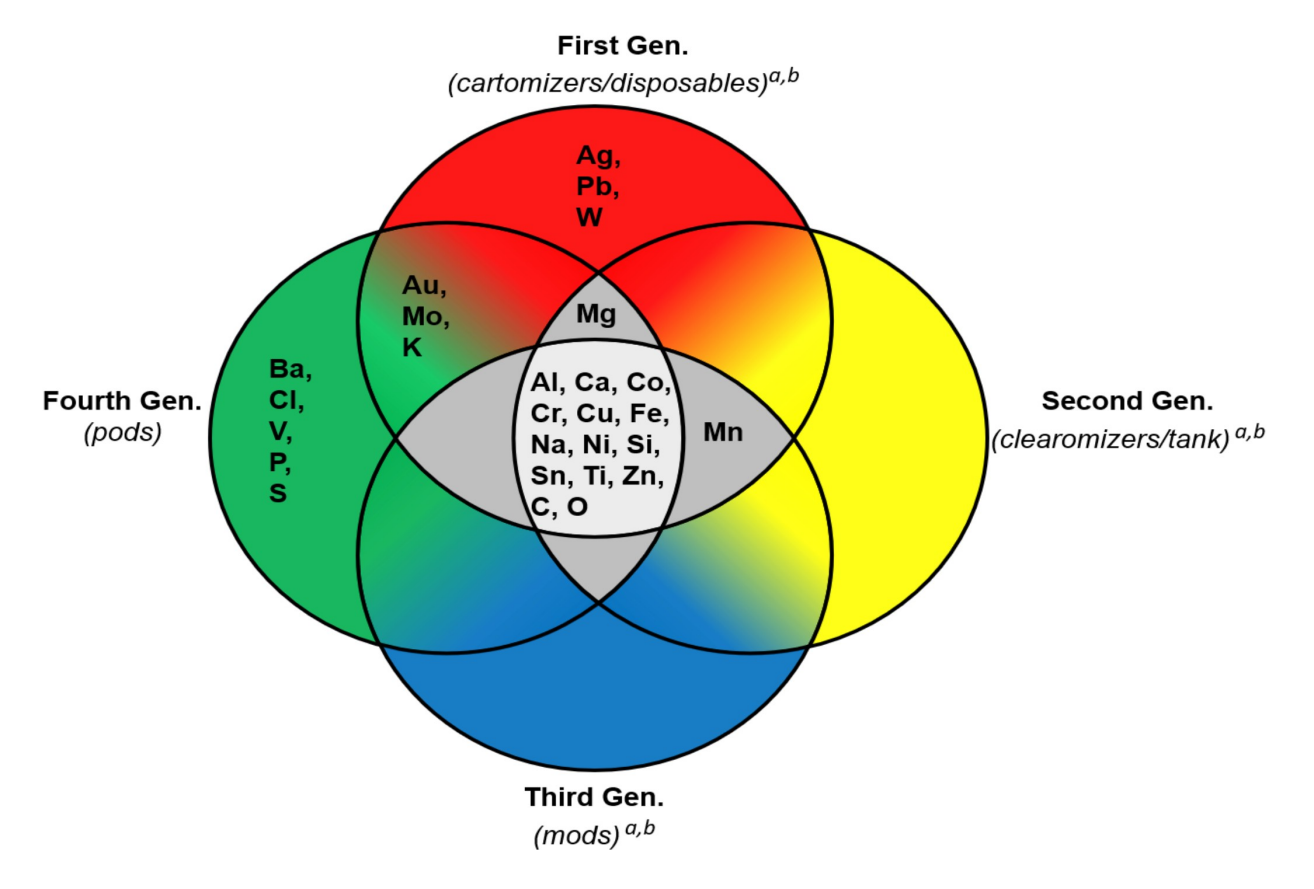
Venn diagram showing elements identified in atomizer components of four EC generations. Al = aluminum, Co = cobalt, Cr = chromium, Cu = copper, Fe = iron, Ni = nickel, Sn = tin, Ti = titanium, Zn = zinc, Ag = silver, Pb = lead, W = tungsten, V = vanadium, Au = gold, Mo = molybdenum, Mn = manganese, Mg = magnesium, C = carbon, Ca = calcium, Na = sodium, O = oxygen, Si = silicon, Ba = barium, Cl = chlorine, P = phosphorus, S = sulfur, K = potassium. ^a^ = Williams et al. 2019a; ^b^ = Williams et al. 2019b.

Fourteen elements (aluminum, calcium, cobalt, chromium, copper, iron, sodium, nickel, silicon, tin, titanium, zinc, carbon, and oxygen) have been identified in atomizer components from all generations (Fig 9). While silver, lead, and tungsten were present only in first-generation products, manganese was found in the first, second, and third generations. Magnesium was present in components of the first, second, and fourth generations. Barium, chlorine, vanadium, phosphorus, and sulfur were only in the fourth generation. However, gold, molybdenum, and potassium were identified in components of first and fourth generation ECs. There were no elements identified in either second or third generations, which were not found in other generations (Fig 9).

## Discussion

Our study compares the design features and elemental composition of atomizers in pod ECs from multiple popular manufacturers. The pod fluid reservoirs were either prefilled (JUUL™, PHIX, and Kilo 1K), prefilled and refillable (KWIT Stick), or refillable SMOK (Infinix, Mico, and NORD) and Suorin (Air, Edge, and Drop). Even though multiple components in the pods were similar (e.g., filament, air tube, wicks, and connector plate/pin), there were variations in fluid reservoirs, battery capacity, and elemental composition across our sample of prefilled and refillable pod components. A total of 23 elements were identified in the EC pod atomizers, of which 11 were considered dominant elements based on their relative abundance in EDS spectra.

Like earlier EC generations, the pod ECs varied in shape and size. The most striking design difference in the pods was the modern and futuristically shaped batteries (S1 Fig) [1, 4]. The pod batteries are smaller than those in clearomizers and mods, but larger than cig-a-likes. EC pod batteries operate at a relatively low fixed voltage, unlike clearomizers/mods that have higher variable voltages with higher potential to release atomizer elements into the aerosol [1].

Some pod atomizer components, such as the filament, thick wire, air tube, wick, and wire-wire joint, were preserved across generations (Fig 8A). Brazing, a wire joining method, and organic wicks were also found in all EC generations. Components such as the gold-plated connectors, connector-wire joint, wick chamber, magnets, and outer casing were observed for the first time in pods (Fig 8A). The insulating sheaths in previous models were absent in the fourth generation. Differences, such as the inclusion of ceramic wicks, connectors, and variations in filaments (coil vs. mesh), were seen in some pod atomizers.

Of 23 elements identified in pod atomizers from different manufacturers, 11 (48%) were present in relatively high abundance (nickel, chromium, iron, gold, copper, zinc, tin, oxygen, silicon, carbon, and sodium). Twelve elements (52%) were present in lesser amounts (aluminum, molybdenum, titanium, sulfur, cobalt, calcium, potassium, phosphorus, vanadium, barium chlorine, and magnesium). Except for gold, which was only present in the first and fourth generations, all the high abundance elements have been identified in previous EC generations. (Fig 9), [6]. While filaments in older EC atomizers were mostly nichrome [6], pod filaments were mainly Elinvar. The elemental composition of the JUUL™ filament and connector plate was in agreement with a previous report [27]. Thick wires, mostly copper and silver [6], have evolved into predominantly nickel. Wire-wire joints, which were previously mainly chromium, copper, nickel, tin, and zinc [6], are often Elinvar or iron-containing alloys. Previously used wire-air-tube joints consisting of mainly tin solder in most first-generation ECs have become obsolete. A new wire-connector joint made mainly of Elinvar is present in the fourth generation. In general, the dominant elements in the air tubes and wicks were similar in all generations.

There are several possible sources for the elements in EC aerosols. Atomizer fluids contain elements/metals prior to heating [13, 15–19, 28]. Some of these elements are likely contaminants of the solvents, flavor chemicals, and nicotine that comprise the fluid. Others may leach into the fluid from the atomizer components. Some pod fluids have a low pH, which may facilitate the transfer of elements into fluids before vaping [10], although, in a recent comparison of fluids with pHs as low as 4.02 and as high as 6.79, high metal concentrations did not correlate with low pH [29]. Elements in EC atomizers can also be released into fluids during heating [18, 19] and then inhaled by users. The concentration of metals in EC aerosols can be manipulated by changing the power at which aerosols are generated and/or altering the metals used in the ECs. For example, there has been a gradual reduction in tin solder joints close to the filament and a corresponding decrease in tin in the aerosols [21]. It was recently suggested, based on single particle inductively coupled plasma mass spectrometry, that steel components in the atomizers, not the nichrome filament, are the source of chromium, iron, and nickel in fourth generation aerosols [30]. The plastic components in pod atomizers (e.g., air tubes and fluid reservoirs) may also leach metals and non-metals, such as plasticizers, into pod fluids, which could contribute to aerosol toxicity.

We evaluated a subset of currently marketed EC pods. Products not evaluated in our study may contain additional elements/metals. Likewise, counterfeit products, which were not included in our study, may differ from those produced by major manufacturers [31].

Metals in e-liquids do transfer into the aerosols of first, second, and third generation products [5, 12, 17–21] and are therefore inhaled by EC users. Metal transfer to aerosols has also recently been shown for fourth generation products, such as myblu™ and Vuse Alto® pods, which had elevated levels of chromium, nickel, copper, zinc, tin, and lead in their aerosols [29, 30]. However, not all pods showed this transfer, e.g., the concentrations of these metals in JUUL™ aerosols were at or below the limit of detection and/or limit of calibration standard [29]. This variability between brands has also been shown for prior generations of ECs [32]. Variations in the liquid-to-aerosol transfer can also occur within pod brands. For example, nickel transferred more efficiently to aerosols made using myblu™ Intense Mint-sation than those made with myblu™ Intense Tobacco Chill [29].

Elements such as arsenic, lead, cadmium chromium, cobalt, nickel, and silica have been linked to human illnesses, including cardiovascular diseases, immune system suppression, lung injury, cancer, renal damage, neurotoxicity, and silicosis [33–42]. The metals in EC products have not yet been directly linked to these illnesses, and such linkage may be challenging to demonstrate given the high variability in metal transfer to EC aerosols from different products and the variations in user topography [43], which also affects metal concentrations in aerosols [44]. A recent risk assessment study based on published concentrations of metals in EC products concluded that nickel and chromium are high enough in EC liquids and aerosol to present a cancer risk and that nickel, chromium, and manganese may also present non-cancer health risks [45]. In addition, human urine samples from EC users had higher concentrations of zinc than those from nonsmokers, and zinc concentration was positively correlated with increased DNA oxidation, suggesting a potential increased risk for disease in the EC user group [46]. Data clearly show that EC liquids and aerosols contain elements/metals known to cause disease with chronic exposure. However, because of the variability between and within EC products, it is difficult for users to identify products that may be safer to use.

In summary, we characterized the design features of pod EC, then mapped 23 elements/metals in the atomizers of pods from six manufacturers. The elements/metals in atomizers are important for two reasons. First, chronic exposure could adversely affect human health. Some of the elements/metals are known to produce disease, although this has not yet been demonstrated for the toxic elements in ECs. Secondly, EC pod products are eventually discarded into the environment, contributing to chemical pollution in water and soil. Understanding the health impact of the elements/metals in EC pods and their fate when discarded will be important when establishing regulations on their use and disposal.

## Supporting information

S1 Fig Prefilled and refillable fourth-generation EC products. (A) Ready to use pod devices with batteries. (B) The fluid reservoir compartment arranged by the brand from left to right is JUUL™, KILO 1K, PHIX, KWIT Stick, SMOK Infinix, SMOK NORD, SMOK Mico, Suorin Drop, Suorin Air, and Suorin Edge.
(PDF)

S2 Fig EDS spectra of JUUL™ atomizer components. (A) The wick, which comprises silicon and oxygen, and (B) The connector plate, mainly nickel coated with gold. The Blue arrow indicates the wick, and the purple arrow indicates the connector plate.
(PDF)

S3 Fig SEM images and EDS maps of pod EC filaments and joints. For filaments, Suorin Air (A) was made of iron (B), chromium (C), nickel (D), molybdenum (E), and silicon (F). Suorin Edge (G) was made of iron (H), chromium (I), nickel (J), and silicon (K). For the wire-wire joints, KWIT Stick (L) was made of nickel (M), chromium (N), titanium (O), and iron (P). For connector-to-wire joints of the Suorin Drop (Q) was made of iron (S) and chromium (U) but not nickel (R) and gold (T).
(PDF)

S4 Fig SEM images and EDS maps of pod EC air tubes and wicks. For the air tubes, JUUL™ (A) also contained vanadium (B). (C) SMOK Mico was made of nickel (D), tin (E), cobalt (F), zinc (G), and sulfur (H). (I) KILO 1K was made of iron (J), chromium (K), and nickel (L). (M) PHIX was made of iron (N), chromium (O), and nickel (P). (Q) SMOK NORD (regular coil) was made of nickel (R), tin (S), cobalt (T), copper (U), zinc (V), and iron (W). The SMOK Mico ceramic wick (X) contained oxygen (Z), silicon (AA), phosphorus (BB), aluminum (CC), potassium (DD), sodium (EE), but not nickel (Y). The KILO 1K organic wick (FF) was silicon and (GG), and oxygen (HH).
(PDF)

S5 Fig SEM images and EDS maps of pod EC connectors. Connectors present in JUUL™ (A) were made of nickel (B), gold (C), iron (D), and chromium (E). PHIX (F) was made of nickel (G), gold (H), and copper (I). SMOK Mico (J) was made of nickel (K), gold (L), copper (M), and zinc (N). SMOK NORD (O) was made of nickel (P), gold (Q), copper (R), zinc, and iron (S). SMOK Infinix (T) was made of nickel (U), gold (W), copper (X), zinc (Y), silicon (Z), and aluminum (AA) but not chromium (V).
(PDF)
