# Supplementary material for: Design features and elemental/metal analysis of the atomizers in pod-style electronic cigarettes
Source: PLoS One. 2021 Mar 9;16(3):e0248127. doi: 10.1371/journal.pone.0248127 (PMC7943009; doi:10.1371/journal.pone.0248127)
Supplement: S1 Fig — (A) Ready to use pod devices with batteries. (B) The fluid reservoir compartment arranged by the brand from left to right is JUUL™, KILO 1K, PHIX, KWIT Stick, SMOK Infinix, SMOK NORD, SMOK Mico, Suorin Drop, Suorin Air, and Suorin Edge. (PDF) [file pone.0248127.s001.pdf]

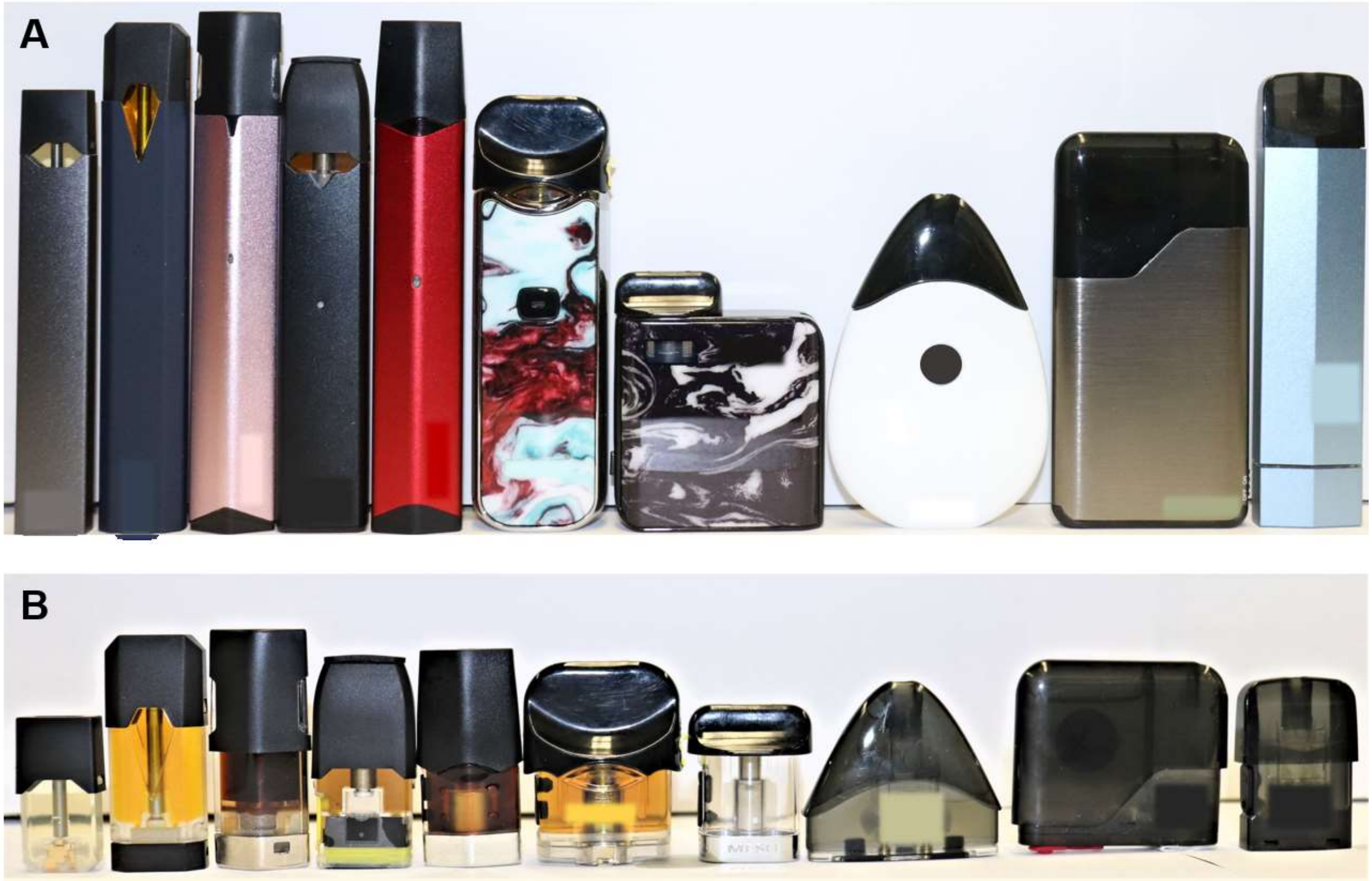

S1 Fig. Fourth-generation pre-filled and refillable EC pod products. (A) ready to use pod devices with batteries, and (B) pods only arranged by brand from left to right as follows; JUUL, KILO 1K, PHIX, KWIT Stick, SMOK Infinix, SMOK NORD, SMOK Mico, Suorin Drop, Suorin Air, and Suorin Edge. (TIF)
