# Supplementary material for: Design features and elemental/metal analysis of the atomizers in pod-style electronic cigarettes
Source: PLoS One. 2021 Mar 9;16(3):e0248127. doi: 10.1371/journal.pone.0248127 (PMC7943009; doi:10.1371/journal.pone.0248127)
Supplement: S2 Fig — (A) The wick, which comprises silicon and oxygen, and (B) The connector plate, mainly nickel coated with gold. The Blue arrow indicates the wick, and the purple arrow indicates the connector plate. (PDF) [file pone.0248127.s002.pdf]

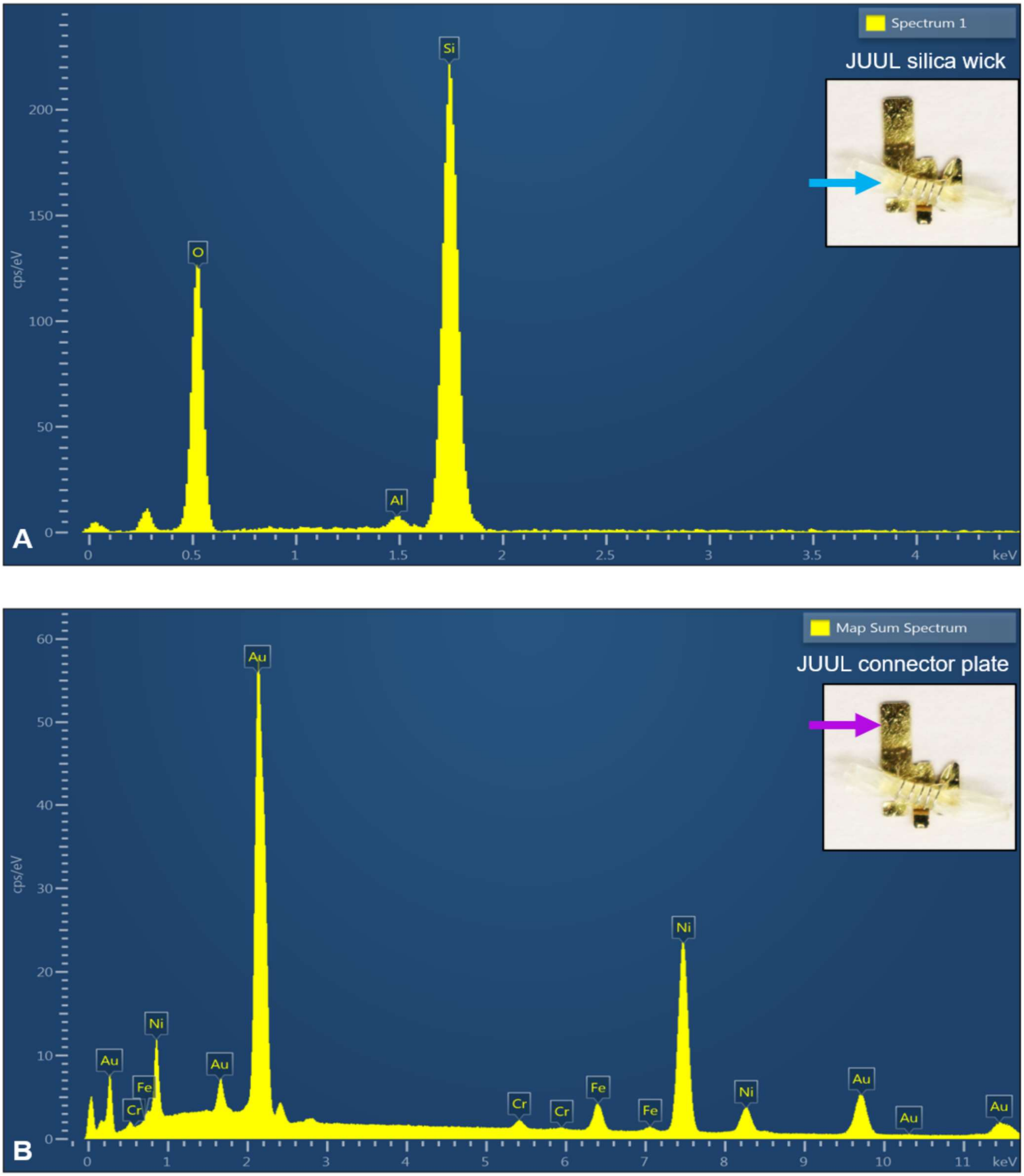

S2 Fig. EDS spectra of JUUL atomizer components. (A) wick, which comprises silicon and oxygen, and (B) the connector plate, mainly nickel coated with gold. The Blue arrow indicates the wick, and the purple arrow indicates the connector plate.
