# Supplementary material for: Design features and elemental/metal analysis of the atomizers in pod-style electronic cigarettes
Source: PLoS One. 2021 Mar 9;16(3):e0248127. doi: 10.1371/journal.pone.0248127 (PMC7943009; doi:10.1371/journal.pone.0248127)
Supplement: S3 Fig — For filaments, Suorin Air (A) was made of iron (B), chromium (C), nickel (D), molybdenum (E), and silicon (F). Suorin Edge (G) was made of iron (H), chromium (I), nickel (J), and silicon (K). For the wire-wire joints, KWIT Stick (L) was made of nickel (M), chromium (N), titanium (O), and iron (P). For connector-to-wire joints of the Suorin Drop (Q) was made of iron (S) and chromium (U) but not nickel (R) and gold (T). (PDF) [file pone.0248127.s003.pdf]

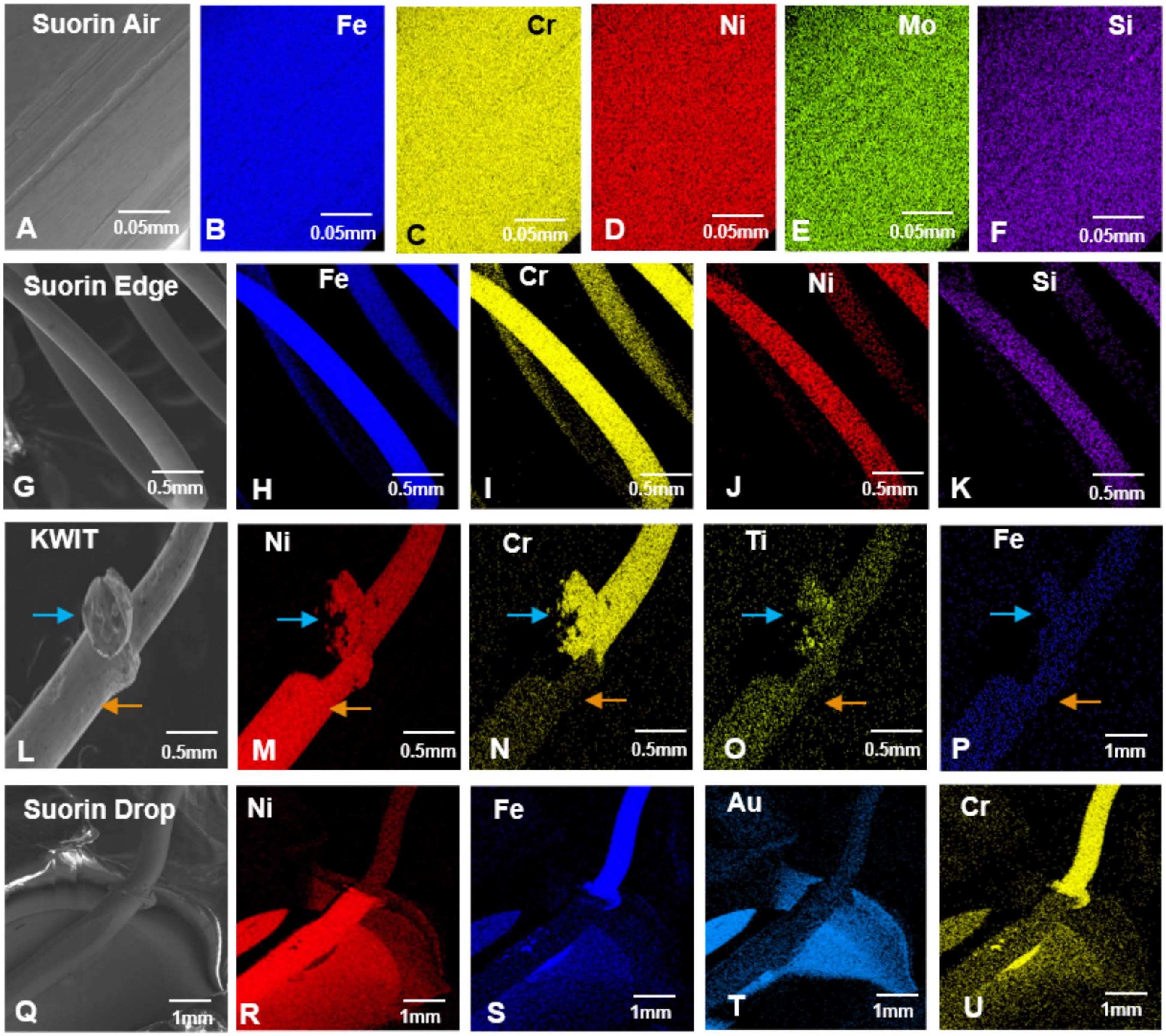

S3 Fig. Scanning electron microscopy images and EDS elemental maps of the filaments and joints. For filaments, Suorin Air (A) was made of iron (B), chromium (C), nickel (D), molybdenum (E), and silicon (F). Suorin Edge (G) was made of iron (H), chromium (I), nickel (J), and silicon (K). For the wire-to-wire joints, KWIT Stick (L) was made of nickel (M), chromium (N), titanium (O), and iron (P). For connector-to-wire joints of the Suorin Drop (Q) was made of iron (S) and chromium (U) but not nickel (R) and gold (T).
