# Supplementary material for: Design features and elemental/metal analysis of the atomizers in pod-style electronic cigarettes
Source: PLoS One. 2021 Mar 9;16(3):e0248127. doi: 10.1371/journal.pone.0248127 (PMC7943009; doi:10.1371/journal.pone.0248127)
Supplement: S4 Fig — For the air tubes, JUUL™ (A) also contained vanadium (B). (C) SMOK Mico was made of nickel (D), tin (E), cobalt (F), zinc (G), and sulfur (H). (I) KILO 1K was made of iron (J), chromium (K), and nickel (L). (M) PHIX was made of iron (N), chromium (O), and nickel (P). (Q) SMOK NORD (regular coil) was made of nickel (R), tin (S), cobalt (T), copper (U), zinc (V), and iron (W). The SMOK Mico ceramic wick (X) contained oxygen (Z), silicon (AA), phosphorus (BB), aluminum (CC), potassium (DD), sodium (EE), but not nickel (Y). The KILO 1K organic wick (FF) was silicon and (GG), and oxygen (HH). (PDF) [file pone.0248127.s004.pdf]

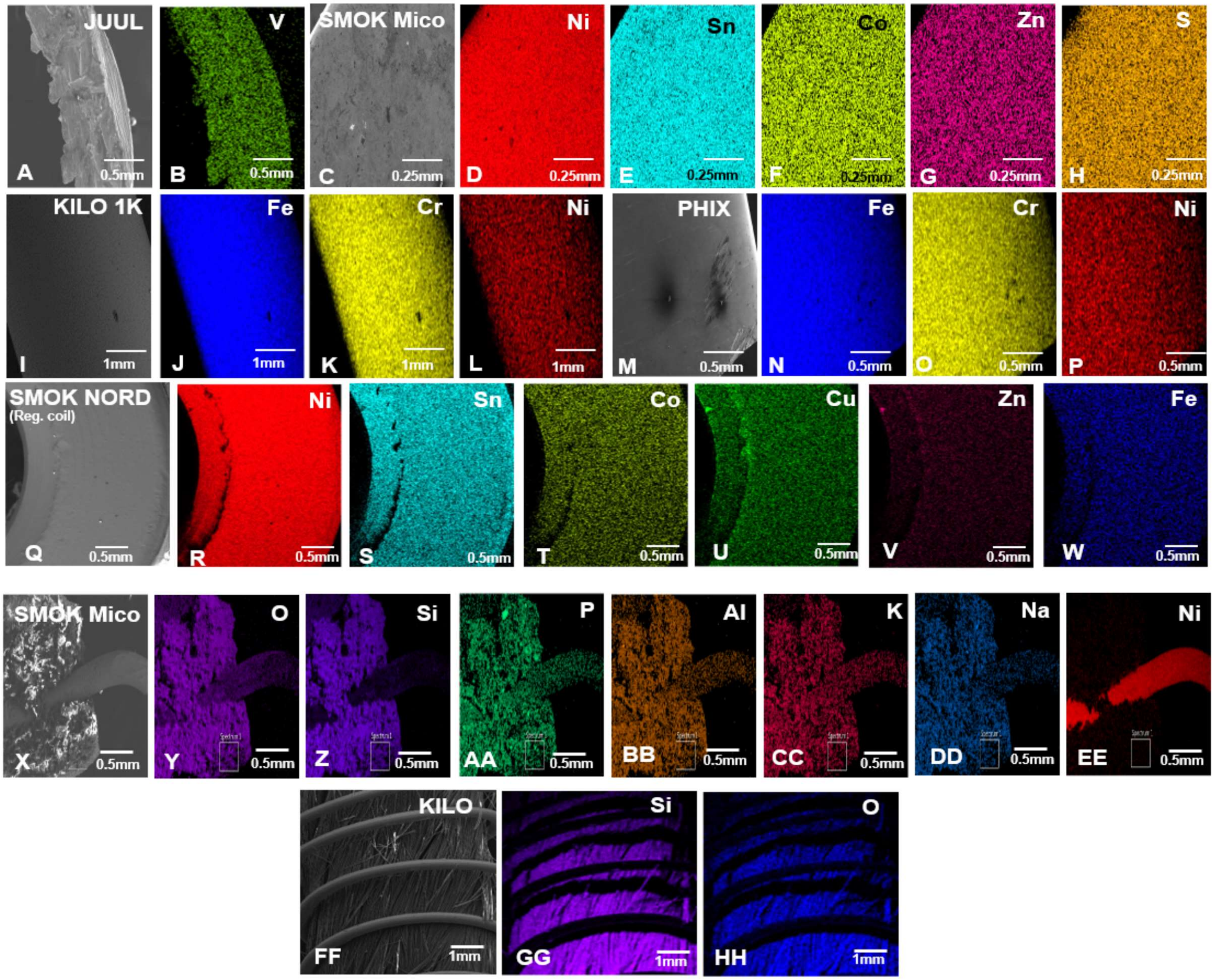

S4 Fig. Scanning electron microscopy images and EDS elemental maps of the air-tubes and wicks. For the air tubes, JUUL (A) also contained vanadium (B). (C) SMOK Mico was made of nickel (D), tin (E), cobalt (F), zinc (G), and sulfur (H). (I) KILO 1K was made of iron (J), chromium (K), and nickel (L). (M) PHIX was made of iron (N), chromium (O), and nickel (P). (Q) SMOK NORD (regular coil) was made of nickel (R), tin (S), cobalt (T), copper (U), zinc (V), and iron (W). The SMOK Mico ceramic wick (X) contained oxygen (Z), silicon (AA), phosphorus (BB), aluminum (CC), potassium (DD), sodium (EE), but not nickel (Y). The KILO 1K organic wick (FF) was silicon and (GG), and oxygen (HH)
