# Supplementary material for: Design features and elemental/metal analysis of the atomizers in pod-style electronic cigarettes
Source: PLoS One. 2021 Mar 9;16(3):e0248127. doi: 10.1371/journal.pone.0248127 (PMC7943009; doi:10.1371/journal.pone.0248127)
Supplement: S5 Fig — Connectors present in JUUL™ (A) were made of nickel (B), gold (C), iron (D), and chromium (E). PHIX (F) was made of nickel (G), gold (H), and copper (I). SMOK Mico (J) was made of nickel (K), gold (L), copper (M), and zinc (N). SMOK NORD (O) was made of nickel (P), gold (Q), copper (R), zinc, and iron (S). SMOK Infinix (T) was made of nickel (U), gold (W), copper (X), zinc (Y), silicon (Z), and aluminum (AA) but not chromium (V). (PDF) [file pone.0248127.s005.pdf]

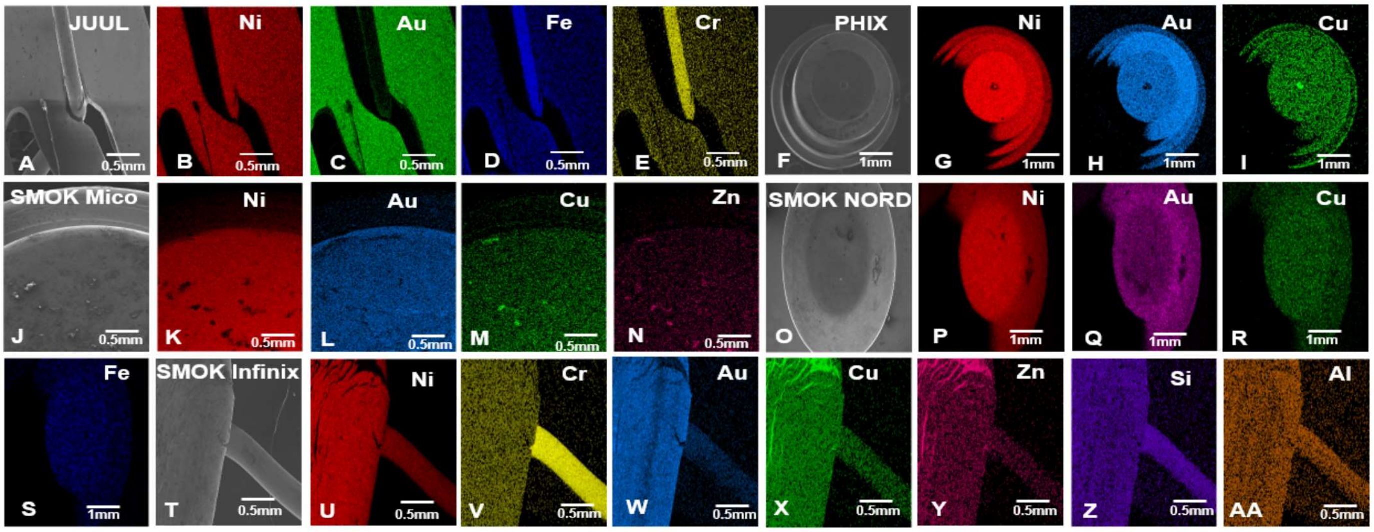

S5 Figure. Scanning electron microscopy images and EDS elemental maps of connector components.

Connectors present in JUUL (A) were made of nickel (B), gold (C), iron (D), and chromium (E). PHIX (F) was made of nickel (G), gold (H), and copper (I). SMOK Mico (J) was made of nickel (K), gold (L), copper (M), and zinc (N). SMOK NORD (O) was made of nickel (P), gold (Q), copper (R), zinc, and iron (S). SMOK Infinix (T) was made of nickel (U), gold (W), copper (X), zinc (Y), silicon (Z), and aluminum (AA) but not chromium (V).
